# Risk Factors for Muscle Loss in Hemodialysis Patients with High Comorbidity

**DOI:** 10.3390/nu12092494

**Published:** 2020-08-19

**Authors:** Wesley J. Visser, Anneke M.E. de Mik-van Egmond, Reinier Timman, David Severs, Ewout J. Hoorn

**Affiliations:** 1 Department of Internal Medicine, Division of Dietetics, Erasmus MC, University Medical Center, 3015 GD Rotterdam, The Netherlands; w.j.visser@erasmusmc.nl (W.J.V.); a.vanegmond@erasmusmc.nl (A.M.E.d.M.-v.E.); 2 Department of Internal Medicine, Erasmus MC, University Medical Center, 3015 GD Rotterdam, The Netherlands; r.timman@erasmusmc.nl; 3 Department of Psychiatry, Erasmus MC, University Medical Center, 3015 GD Rotterdam, The Netherlands; 4 Department of Internal Medicine, Division of Nephrology and Transplantation, Erasmus MC, University Medical Center, 3015 GD Rotterdam, The Netherlands; d.severs@erasmusmc.nl

**Keywords:** hemodialysis, body composition, nutritional status, lean tissue mass, protein-energy wasting

## Abstract

With expanding kidney transplantation programs, remaining hemodialysis patients are more likely to have a high comorbidity burden and may therefore be more prone to lose muscle mass. Our aim was to analyze risk factors for muscle loss in hemodialysis patients with high comorbidity. Fifty-four chronic hemodialysis patients (Charlson Comorbidity Index 9.0 ± 3.4) were followed for 20 weeks using 4-weekly measurements of lean tissue mass, intracellular water, and body cell mass (proxies for muscle mass), handgrip strength (HGS), and biochemical parameters. Mixed models were used to analyze covariate effects on LTM. LTM (−6.4 kg, interquartile range [IQR] −8.1 to −4.8), HGS (−1.9 kg, IQR −3.1 to −0.7), intracellular water (−2.11 L, IQR −2.9 to −1.4) and body cell mass (−4.30 kg, IQR −5.9 to −2.9) decreased in all patients. Conversely, adipose tissue mass increased (4.5 kg, IQR 2.7 to 6.2), resulting in no significant change in body weight (−0.5 kg, IQR −1.0 to 0.1). Independent risk factors for LTM loss over time were male sex (−0.26 kg/week, 95% CI −0.33 to −0.19), C-reactive protein above median (−0.1 kg/week, 95% CI −0.2 to −0.001), and baseline lean tissue index ≥10th percentile (−1.6 kg/week, 95% CI −2.1 to −1.0). Age, dialysis vintage, serum albumin, comorbidity index, and diabetes did not significantly affect LTM loss over time. In this cohort with high comorbidity, we found universal and prominent muscle loss, which was further accelerated by male sex and inflammation. Stable body weight may mask muscle loss because of concurrent fat gain. Our data emphasize the need to assess body composition in all hemodialysis patients and call for studies to analyze whether intervention with nutrition or exercise may curtail muscle loss in the most vulnerable hemodialysis patients.

## 1. Introduction

In patients with chronic kidney disease (CKD) who undergo chronic hemodialysis, nutritional status, body composition, and especially muscle mass, are closely linked to morbidity, mortality, and quality of life [1,2,3]. There are multiple factors that lead to muscle mass loss in patients undergoing hemodialysis. First, with progression of CKD, there is a decline in protein intake [4], and anorexia is reported in approximately one-third of hemodialysis patients [5]. Second, fluid restriction may lead to a concurrent decrease in caloric intake [6]. Third, the hemodialysis procedure itself may contribute to the catabolic state due to decreased protein synthesis and increased proteolysis [7,8]. Finally, additional catabolic factors may be present that lead to muscle mass loss in hemodialysis patients including acidosis, comorbidity, inflammation, corticosteroid use, and sedentary lifestyle [9,10].

With expanding kidney transplantation programs, remaining hemodialysis patients are more likely to have a high comorbidity burden and may therefore be more prone to lose muscle mass. Therefore, we hypothesized that loss of muscle mass and muscle strength is especially prominent in hemodialysis patients with a high comorbidity burden. The Netherlands has a high kidney transplantation rate per million population [11]. Our university hospital harbors a large kidney transplantation program (approximately 200 transplantations per year). Accordingly, a relatively large proportion of our in-center hemodialysis population cannot be transplanted because of comorbidity. We considered that this specific hemodialysis population was suitable to address our hypothesis. Indeed, in this prospective longitudinal study, we find a dramatic and universal loss of both muscle mass and muscle strength in hemodialysis patients with high comorbidity. This particular group may be especially suited for interventions with nutrition or exercise.

## 2. Materials and Methods

### 2.1. Study Design and Subjects

The study protocol was reviewed and approved by our medical ethical review board (MEC-2017-445). We prospectively included adult patients undergoing chronic in-center hemodialysis from September to December 2017. All patients were included except if they had specific exclusion criteria, including life expectancy ≤6 months, active treatment for malignancy or infection, a (unipolar) pacemaker, and the use of intradialytic parenteral nutrition (IDPN). The first measurement of this study, regardless of dialysis vintage, was defined as the baseline measurement. Patients were followed for a minimum of 3 to a maximum of 6 measurements with 4-weekly study visits. Our standard of care includes a target spKt/V >1.4 and predialysis plasma bicarbonate >22 mmol/L. Dietary advice and support were provided to all subjects as standard policy. Dietary counseling includes an advice for protein requirement in the range of 1.0–1.2 g/kg and for patients with malnutrition or inflammation 1.2–1.5 g/kg and a calculation of the individual energy requirement by adding 30% to the estimated resting energy expenditure [12]. For all patients who did not meet their nutritional requirements, sip feeding, tube feeding and/or intradialytic parenteral nutrition is a possible treatment. During dialysis, we offer all patients energy and protein-rich food.

### 2.2. Measurements

Body composition was assessed with the Body Composition Monitor (Fresenius Medical Care, Bad Homburg, Germany), which is based on bio-impedance spectroscopy (BIS) at 50 different frequencies ranging between 5 and 1000 kHz. The Body Composition Monitor has been validated against gold-standard reference methods [13] and has the ability to differentiate between excess fluid and normally hydrated lean tissue mass [14]. The following parameters were generated during each measurement: total body water (TBW), extracellular water (ECW), intracellular water (ICW), lean tissue mass (LTM), body cell mass (BCM), adipose tissue mass (ATM), phase angle, and estimated predialysis overhydration. Lean tissue index (LTI) and fat tissue index (FTI) were calculated respectively as LTM and ATM divided by height^2^ (kg/m^2^) and compared with reference values (10th percentile) for age and gender [15]. Phase angle is an measure related to body cell mass and the ratio between extracellular and intracellular fluid, and is calculated as the arc tangent of reactance over resistance. BIS measurements were performed using a standardized protocol and experienced operators before the start of the dialysis session. Dry weight was recorded from the dialysis prescription most recent from each study visit. Physical function was assessed by handgrip strength measured with a hand dynamometer (hydraulic, JAMAR; Patterson Medical, Warrenville, IL, USA). The test was performed in a sitting position, with the patient instructed to perform three consecutive contractions with both hands (except for AV fistula side). The highest value was compared with reference values [16]. Protein intake was estimated by the normalized protein catabolic rate (nPCR) [17]. The nPCR is based on interdialytic (ID) changes in blood urea nitrogen (BUN) concentrations and urinary protein and urea excretion, where nPCR in g/kg per day = 0.22 + (0.036 × ID rise in BUN × 24)/ID interval (hours). In patients with urine output ≥200 mL/day, we added the following calculation to the equation: urinary urea nitrogen (g) × 150/ID interval (hours) × weight (kg) [17]. Serum albumin (bromocresol green method), and serum C-reactive protein (CRP) were measured using the Cobas 8000 modular analyzer series (Roche Diagnostics, Almere, The Netherlands). Blood samples were taken every four weeks before dialysis. For assessing comorbidity, we used the Charlson Comorbidity Index [18].

### 2.3. Statistical Analysis

**Figure 1:**
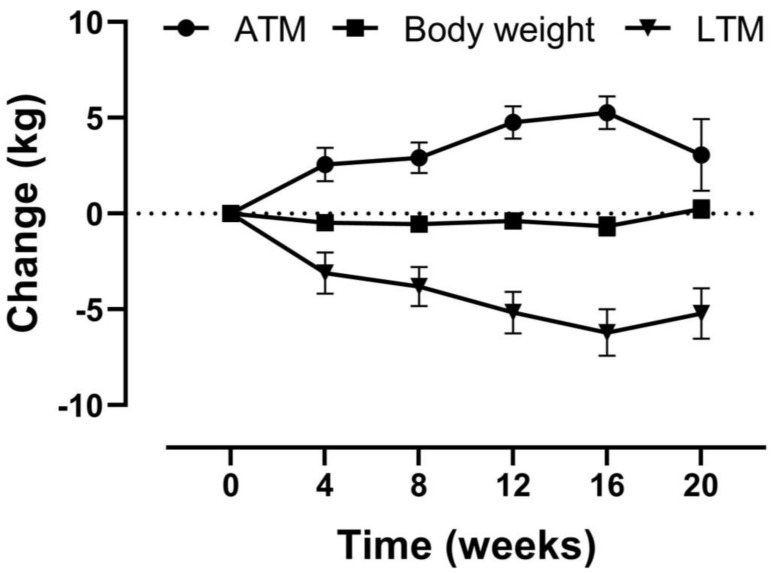
Change in body weight, lean tissue mass (LTM), and adipose tissue mass (ATM) over time. Data are shown as the mean ± SEM. Measurements available for all 54 patients (t = 0, 4 and 8 weeks), 46 patients (t = 12 weeks), 42 patients (t = 16 weeks), and 37 patients (t = 20 weeks).

The primary endpoint was LTM, which was measured 3 to 6 times during this study. For the longitudinal analyses, mixed models were applied. The results from the mixed models analyses are reported in the tables, while recorded data are shown in Figure 1. Two levels were included in the mixed models, of which the upper level represented the patients and the lower level their repeated measures. Time was postulated as a continuous linear fixed effect. The covariance structure was determined with the deviance statistic [19] using restricted maximum likelihood [20]. The statistical analyses for the secondary study parameters were the same as for the primary endpoint. For the exploration of potential influences of the covariates, the multilevel analyses were extended with these effects and their interactions with time as covariates. These covariates were gender, age < or ≥65 years, LTI < or ≥10th percentile, dialysis vintage < or ≥12 months, CCI < or ≥mean, serum CRP and albumin levels below or above median (all as dichotomous variables). Dropout analyses were performed with independent group *t*-tests by comparing baseline LTM measures between the retained and dropped-out patients at 20 weeks. *p*-values < 0.05 were considered statistically significant. Statistical analyses were performed with IBM-SPSS version 24.

## 3. Results

### 3.1. Baseline Characteristics

Sixty-four hemodialysis patients were screened for inclusion, of whom 54 were included. Reasons for exclusion were a unipolar pacemaker (*n* = 1), active treatment for malignancy (*n* = 2) or infection (*n* = 2), and the use of intradialytic parenteral nutrition (*n* = 5). Baseline characteristics are shown in Table 1. Our cohort was relatively old, consisted predominantly of males, and had a high prevalence of diabetes and cardiovascular disease. This resulted in a high Charlson Comorbidity Index (9.0 ± 3.4). All 54 included patients were followed up for at least 8 weeks (3 study visits), while 37 patients completed the 20 week visit (6 study visits). There was no significant difference in baseline LTM between patients with three and patients with six measurements (39.8 vs. 37.1 kg; *p* = 0.5). Loss to follow up was uniformly caused by transfer to other dialysis centers.

### 3.2. Lean Tissue Mass Is Replaced by Adipose Tissue Mass

Body weight did not decrease significantly in 20 weeks (−0.5 kg, 95% confidence interval [CI] −1.0 to 0.1), which was caused by a decrease in LTM (−6.4 kg, 95% CI −8.1 to −4.8) and an increase in ATM (4.5 kg, 95% CI 2.7 to 6.2, Table 2 and Figure 1). In addition to LTM, ICW (−2.11 L, 95% CI −2.9 to −1.4) and BCM (−4.30 kg, 95% CI −5.9 to −2.9) also decreased significantly (Table 2). Handgrip strength decreased by −1.9 kg (95% CI −3.1 to −0.7). There were no significant changes over time in phase angle, predialysis overhydration, nPCR, serum albumin, and serum CRP (Table 2). In addition, no significant changes were observed in target dry weight and body mass index (data not shown). A sensitivity analysis was performed including only those participants with complete follow up, which showed similar results (Table S1).

### 3.3. Male Sex and Inflammation Accelerate Loss of Lean Tissue Mass

All patients demonstrated a significant loss of LTM over time, regardless of stratifying the covariates age, sex, LTI, baseline serum CRP, serum albumin, dialysis vintage, diabetes, or comorbidity index (Table 3). However, LTM loss over time was greater in males and patients with higher baseline CRP and LTI. These covariates remained independent predictors of greater LTM loss in the multivariate analysis (Table 4). The presence of diabetes, lower serum albumin, longer dialysis vintage, and higher comorbidity index were not associated with greater LTM loss over time.

## 4. Discussion

The aim of this prospective and longitudinal study was to identify risk factors for muscle loss in chronic hemodialysis patients with high comorbidity. To do so, we performed serial measurements of lean tissue mass (LTM) as a proxy for muscle mass. The results show that all patients experienced muscle loss and that on average this loss was very pronounced (−6.4 kg in 20 weeks or ~1.3 kg/month). Muscle loss was further accelerated by male sex and by inflammation. Because we focused specifically on hemodialysis patients with high comorbidity, the magnitude of the changes in body composition was considerably greater in this study than in most previous studies (summarized in Table 5). We also show that muscle loss was not reflected by a change in body weight or body mass index, because of a concurrent gain in fat mass. This is the first study to analyze muscle loss in hemodialysis patients with high comorbidity and our results suggest that this specific patient category may benefit from nutritional or exercise interventions. Our study also reinforces the need to more routinely perform body composition monitoring to estimate muscle mass. Additional strengths of this study are the inclusion of patients regardless of dialysis vintage and the inclusion of handgrip strength, a measure of muscle function that correlates well with physical function and all-cause mortality [21,22].

The link between inflammation and muscle loss that we identified in this study has been reported previously [32]. Inflammation may decrease protein anabolism, increase protein catabolism, or both [33]. A state of low-grade inflammation, as indicated by elevated CRP or other acute-phase reactants, is highly common in patients undergoing dialysis treatment [34]. Remarkably, this effect on LTM loss has not previously been demonstrated in longitudinal studies in hemodialysis patients, although Johansen et al. did report an association between higher interleukin 1β concentrations [29]. Patients in our study had a higher CRP than in most previous reports (Table 5) [24,28,29]. In addition to CRP, serum albumin correlates with LTM in chronic kidney disease patients [35]. Moreover, serum albumin usually inversely correlates with inflammatory parameters [36]. Our failure to detect this effect, as well as the relatively high serum albumin concentrations in our population, may partly be explained by the measurement method we employed. The bromocresol green assay used in this study significantly overestimates serum albumin concentrations when compared with reference immunoassays [37]. Importantly, interference by acute-phase reactants is partially responsible for this error [38], which could have led to an underestimation of the effect of serum albumin concentration on LTM loss in our study.

The observation that males on hemodialysis have a greater loss of muscle mass than females was also reported by Marcelli et al. [24] and may be related to the response to inflammation [39]. For example, male but not female dialysis patients with inflammation have worse outcomes [40]. There are clear sex differences in skeletal muscle kinetics and fiber-type composition [41]. In addition, there are several theoretical explanations for sex differences in muscle wasting, including the anabolic effects of testosterone, and the anti-inflammatory effects of estrogen [42,43]. Testosterone deficiency is especially common in male dialysis patients with low muscle mass [44] and this may have contributed to the greater LTM loss over time. Conversely, estrogen may have exerted an anti-inflammatory effect in the premenopausal women included in this study.

Three other observations in this study merit discussion. First, we found that patients with LTI > P10 at baseline were also more prone for LTM loss over time. This seems logical, because these patients “have more to lose”. Yet, this finding is clinically relevant, because patients with higher LTM at baseline will likely not be selected for close monitoring of nutritional status. The need for monitoring of nutritional status was also illustrated by the fact that the baseline BMI was 25.6 kg/m^2^, whereas 76% of the patients had some degree of malnutrition according to SGA and 48% had an LTI < P10. Second, the rate of LTM loss over time was not significantly influenced by the presence of diabetes mellitus, which differs from the study by Pupim et al. [28]. Interestingly, in that study, patients with diabetes had a significantly higher baseline LTM than patients without diabetes, while we observed the opposite in our cohort. This may be explained by differences in study population, measurement methods (DEXA vs. BIS), or dialysis vintage. Third, in our study the magnitude of LTM loss was not significantly affected by dialysis vintage. The observation that “stable” hemodialysis patients may still lose significant muscle mass corresponds with findings by Molina et al., who reported a mean 12-month decrease in LTM of 6.8 kg in a population with a median dialysis vintage of 40 months [23]. In contrast, Chertow et al. found only a small negative effect of vintage on body cell mass in a large patient sample [45]. Importantly, cross-sectional analyses such as theirs probably underestimate changes in body composition. We argue that factors that precipitate loss of muscle mass likely exist irrespective of dialysis vintage.

This study has a number of limitations. First, this is a single-center observational study with relatively short follow up, and therefore the results may not be generalizable to other settings. However, our specific aim was to address muscle loss in hemodialysis patients with high comorbidity. Therefore, our specific hypothesis was addressable in this specific patient population whose Charlson Comorbidity Index was clearly higher than in previous studies [46,47,48]. A second limitation may be that not all patients completed all visits and that the sample size was moderate. However, we applied specific statistical methodology to account for missing data (mixed models) and the sample size was comparable to previous prospective studies (Table 5). A third limitation may be that we measured body composition before dialysis. This was done to allow assessment of predialysis overhydration, ultrafiltration rate, and target dry weight. In addition to LTM, we recorded ICW and BCM as proxies of muscle mass, as recommended by Carrero et al. when assessing body composition before dialysis [49]. These data support the LTM results and confirm that the observed changes indeed reflect loss of muscle mass. Finally, protein intake in our cohort was slightly below recommendations, but similar to previous studies [50]. In addition, we achieved this protein intake by following current guidelines for nutrition, suggesting that other approaches such as IDPN may be necessary to meet the recommendations [51].

In summary, in this prospective longitudinal study in chronic hemodialysis patients, we found a major loss in LTM. A simultaneous increase in ATM obscured changes in body weight. This observation was most pronounced in males and in patients with evidence of inflammation and was present regardless of dialysis vintage. Muscle strength at baseline was low and decreased significantly during this study. The findings of this study demonstrate the relevance of monitoring body composition in all hemodialysis patients. Coupled with the growing amount of data supporting a link between muscle mass and clinical outcomes in dialysis patients [1,2,3], our data call for studies to analyze whether exercise or nutritional interventions, such as oral nutritional supplements or IDPN, are able to maintain or increase LTM and muscle strength.

## Figures and Tables

**Table 1:** Baseline characteristics of study cohort (*n* = 54).

General Characteristics	Age (Years)	66 (52, 74)
	Males, *n* (%)	38 (71)
Comorbidities	Charlson Comorbidity Index	9.0 ± 3.4
	Diabetes, *n* (%)	20 (37)
	Cardiovascular disease, *n* (%)	38 (71)
Dialysis characteristics	Dialysis vintage (months)	19 (7, 40)
	spKt/V	1.49 ± 0.24
Laboratory values	Albumin, g/L	38 (36.6, 39.2)
	C-reactive protein, mg/L	11 (7.6, 14.3)
Body composition	Body weight, kg	76.7 (71.9, 81.6)
	Body mass index for dry weight, kg/m^2^	25.6 (22.3, 28.5)
	Predialysis overhydration (mL)	1278 (835, 1712)
	Total body water (L)	34.5 (29.1, 40.5)
	Extracellular water	16.6 (14.5, 19.3)
	Intracellular water	17.5 (14.1, 21.3)
	Body cell mass (kg)	18.3 (12.6, 23.7)
	Lean tissue mass, kg	37.7 (34.7, 40.6)
	Lean tissue index < P10, *n* (%)	26 (48)
	Adipose tissue mass, kg	37.6 (32.6, 42.5)
	Fat tissue index > P90, *n* (%)	14 (26)
	Phase angle,°	4.35 ± 1.1
Muscle strength	Handgrip strength, kg	23 (19.8, 25.2)
	Handgrip strength < P10, *n* (%)	33 (61)
Protein intake	Normalized protein catabolic rate, g/kg	0.9 ± 0.2
Subjective Global Assessment	Severely malnourished (1–2), *n* (%)	1 (2)
	Moderately malnourished (3–5), *n* (%)	40 (74)
	Well nourished (6–7), *n* (%)	13 (24)

Footnote: The data are shown as the mean ± SD or median [IQRs].

**Table 2:** Change in primary and secondary study parameters from mixed models analysis *.

	Baseline	20 Weeks	Difference in Time		
				%	*p*−Value
Lean tissue mass, kg	37.7 (34.7, 40.6)	31.3 (28.2, 34.3)	−6.4 (−8.1, −4.8)	−17.1	<0.001
Body weight, kg	76.7 (71.9, 81.6)	76.2 (71.4, 81.1)	−0.5 (−1.0, 0.1)	−0.6	0.09
Adipose tissue mass, kg	37.6 (32.6, 42.5)	42.1 (37.1, 47.07)	4.5 (2.7, 6.2)	11.9	<0.001
Handgrip strength, kg	22.5 (19.8, 25.2)	20.6 (17.8, 23.3)	−1.9 (−3.1, −0.7)	−8.6	0.002
Pre−dialysis OH, mL	1278 (835, 1712)	1509 (1031, 1973)	236 (−205, 678)	18.5	0.292
Total body water, L	34.5 (29.1, 40.5)	31.7 (28.9, 33.9)	−2.79 (−3.5, −2.0)	−8.1	<0.001
Extracellular water, L	16.6 (14.5, 19.3)	15.9 (14.7, 17.0)	−0.69 (−1.4, −0.5)	−4.3	<0.001
Intracellular water, L	17.5 (14.1, 21.3)	15.4 (13.8, 16.8)	−2.11 (−2.9, −1.4)	−12	<0.001
Body cell mass, kg	18.3 (12.6, 23.7)	14.0 (12.4, 17.4)	−4.30 (−5.9, −2.9)	−23.5	<0.001
Phase angle, °	4.35 (4.04, 4.66)	4.21 (3.89, 4.53)	−0.14 (−0.28, −0.01)	−3.2	0.07
Serum albumin, g/L	38.0 (36.6, 39.2)	37.2 (35.7, 38.4)	−0.8 (−2.0, 0.3)	−2.2	0.1
Serum CRP, mg/L	11 (7.6, 14.3)	9.7 (6.0, 13.21)	−1.3 (−5.5, 2.8)	−12.3	0.5
nPCR, g/kg	0.9 (0.77, 1.03)	1.0 (0.82, 1.09)	+0.1 (−0.05, 0.16)	11.1	0.4

Footnote: * The data are shown as the median [IQRs]. Abbreviations: OH, overhydration; CRP, C-reactive protein; nPCR, normalized protein catabolic rate.

**Table 3:** Lean tissue mass at baseline and after 20 weeks and the effect of covariates.

		Baseline		20 Weeks	Difference		
		kg	*p*-Value	kg	kg	%	*p-*Value
Age	<65 years	41.9		36.1	−5.8	−13.7	<0.001
	≥65 years	33.7		26.8	−6.9	−20.5	<0.001
	difference	8.2	0.004	9.3	1.1	6.8	0.5
Sex	male	42.3		34.9	−7.4	−17.5	<0.001
	female	26.8		22.6	−4.2	−15.7	<0.001
	difference	15.5	<0.001	12.3	3.2	−1.8	0.07
LTI (percentile)	<P10	37.0		31.2	−5.8	−15.6	<0.001
	≥P10	39.3		32.1	−7.2	−18.4	<0.001
	difference	−2.3	<0.001	−0.9	−1.4	−2.8	<0.001
C-reactive protein	<median	37.1		31.6	−5.5	−14.7	<0.001
	≥median	40.0		29.7	−10.3	−25.6	<0.001
	difference	2.9	0.03	−1.9	−4.8	−10.9	0.005
Serum albumin	<median	38.9		31.5	−7.4	−18.9	<0.001
	≥median	37.1		30.9	−6.2	−16.8	< 0.001
	difference	1.8	0.1	0.6	−1.2	−2.1	0.5
Dialysis vintage	<12 months	41.1		32.8	−8.3	−20.2	<0.001
	≥12 months	35.6		30.0	−5.6	−15.8	<0.001
	difference	5.5	0.07	2.8	−2.7	−4.4	0.1
Diabetes mellitus	no diabetes	39.7		34.1	−5.6	−14.1	<0.001
	diabetes	34.2		26.3	−7.9	−23.3	<0.001
	difference	5.5	0.07	7.8	−2.3	−9.2	0.2
Charlson Comorbidity Index	<mean	42.6		36.2	−6.4	−15.1	<0.001
	≥mean	32.8		26.3	−6.5	−19.6	<0.001
	difference	9.8	<0.001	9.9	0.1	4.5	0.9

**Table 4:** Multivariate model for relation of covariates and lean tissue mass.

	Effect ^1^		Time × Effect ^2^	
	Estimate (95% CI)	*p-*Value	Estimate (95% CI)	*p-*Value
Intercept	46.7 (43.4, 49.9)	<0.001	−5.2 (−9.1, −1.4)	0.007
Age ≥ 65 years	−8.4 (−11.8, −4.9)	<0.001	0.595 (−3.2, 4.3)	0.8
Male gender	12.4 (11.6, 13.3)	<0.001	−5.2 (−6.6, −3.8)	0.02
LTI ≥ P10	2.6 (2.1, 3.1)	<0.001	−1.6 (−2.1. −1.0)	<0.001
Serum CRP ≥ median	0.08 (0.004, 0.1)	0.04	−0.1 (−0.2, 0.001)	0.03
Serum albumin < median	−0.08 (−2.3, 2.1)	0.9	0.1 (−3.3, 3.6)	0.9
Dialysis vintage ≥ 12 M	−2.6 (−5.8, 0.07)	0.1	1.5 (−2.3, 5.2)	0.5
Diabetes mellitus	−1.9 (−5.1, 1.3)	0.3	−1.5 (−4.9, 1.9)	0.4
CCI ≥ mean	−0.141 (−3.7, 3.4)	0.9	−3.5 (−7.6, 0.6)	0.09

Footnotes: ^1^ Effect is the effect at baseline, ^2^ Time × effect is for the effect on change in time. Abbreviations: CCI, Charlson Comorbidity Index; CRP, C-reactive protein; LTI, lean tissue index; M, months.

**Table 5:** Overview of studies on change in body composition over time in dialysis patients *.

1st Auth.	Visser	Molina	Marcelli	Keane	Mathew	Di Goia	Pupim	Johansen	Vendrely	Ishimura
**Year**	2020	2018	2016	2016	2014	2012	2005	2003	2003	2001
**Ref.**	-	[23]	[24]	[25]	[26]	[27]	[28]	[29]	[30]	[31]
**Number**	54	32	8227	299	99	84	142	54	30	72
**Design**	P	P	R	R	P	P	P	P	P	R
**Mod.**	HD	HD	HD	HD	HD + PD	HD + PD	HD	HD	HD	HD
**Vintage**	Any	Any	Start	Start	Start	Any	Start	Any	Start	Start
**Age**	66	60	61	63	55	57	53	51	58	62
**%Male**	71	58	64	62	78	34	63	67	66	59
**%DM**	37	18	32	42	39	-	31	-	10	50
**%CVD**	70	21	52	-	-	-	33	-	-	-
**F-U**	20 w	1 y	2 y	2 y	2 y	6 m	1 y	1 y	1 y	1 y
**Method**	BIS	BIS	BIS	BIS	BIS	BIS	DEXA	DEXA	DEXA	DEXA
LTM **	−6.4	−0.5 vs. −6.8	−1.2	−0.9	−1.8	−0.2	−3.4 vs. −1.1	+1.1	0	−0.7
**ATM**	+4.5	−0.1 + 9.8	+2.6	+0.7	+0.6	+0.3	+1.6	−0.4	+2.4	+1.3
**CRP**	11	3.3	6	-	-	5.2	<10	20	-	-
**Albumin**	38	39	38	-	36	37.6	32.5	39	38.5	39

Footnotes: * Age in years, LTM and ATM in kg, CRP in mg/L, albumin in g/L; ** The studies by Molina et al. and Pupim et al. compared the change in LTM in patients receiving hemodiafiltration vs. hemodialysis and in hemodialysis patients with and without diabetes, respectively. Abbreviations: ATM, adipose tissue mass; BIS, bio-impedance spectroscopy; CRP, C-reactive protein; CVD, cardiovascular disease; DEXA, dual energy X-ray absorptiometry; DM, diabetes mellitus; F-U, follow up; HD, hemodialysis; LTM, lean tissue mass; Mod., modality; nPCR, normalized protein catabolic rate; P, prospective; PD, peritoneal dialysis; R, retrospective; Ref., reference.

## Supplementary Materials

The following are available online at https://www.mdpi.com/2072-6643/12/9/2494/s1, Table S1: Change in primary and secondary study parameters for the patients who completed the 20 week visit (*n* = 37).
