# Supplementary material for: Risk Factors for Muscle Loss in Hemodialysis Patients with High Comorbidity
Source: Nutrients. 2020 Aug 19;12(9):2494. doi: 10.3390/nu12092494 (PMC7551970; doi:10.3390/nu12092494)
Supplement: Supplementary file 1 [file nutrients-12-02494-s001.pdf]

Supplementary Materials

Table S1. Change in primary and secondary study parameters for the patients who completed the 20 week visit *(n*=37).

	Baseline	20 weeks	Difference in time		
				%	*p*-Value
Lean tissue mass, kg	37.1 (33.4, 41.0)	31.8 (28.1, 35.7)	-5.3 (-7.2, -3.4)	-14.3	<0.001
Body weight, kg	73.9 (68.7, 79.2)	74.3 (68.6, 82.1)	0.4 (-0.7, 1.0)	0.5	0.09
Adipose tissue mass, kg	36.1 (30.6, 41.5)	40.1 (34.1, 45.6)	4.0 (1.9, 6.0)	11.0	<0.001
Handgrip strength, kg	23.6 (19.6, 27.1)	21.2 (15.9, 25.9)	-2.4 (-3.6, -1.3)	-10.2	<001
Pre-dialysis OH, ml	941 (497, 1386)	1192 (748,1637)	251 (-138, 642)	26.7	0.2
Total body water, L	34.9 (32.5, 37.4)	33.0 (29.7, 36.2)	-1.9 (-2.7, -1.3)	-5.4	<0.001
Extracellular water, L	16.7 (15.6, 17.8)	16.3 (14.9, 17.3)	-0.4 (-0.7,-0.05)	-2.4	0.03
Intracellular water, L	18.3 (16.8, 19.8)	16.7 (14.6, 18.6)	-1.6 (-2.1, -1.0)	-8.7	<0.001
Body cell mass, kg	19.6 (17.2, 22.1)	16.2 (12.7, 19.9)	-3.4 (-4.6, -2.3)	-11.7	<0.001
Phase angle, °	4.58 (4.18, 4.99)	4.35 (3.79, 4.60)	-0.23 (-0.39, -0.07)	-5.0	0.05
Serum albumin, g/L	39.6 (38.1, 41.1)	38.2 (35.6, 40.8)	-1.4 (-2.5, -0.3)	-3.5	0.01
Serum CRP, mg/L	7.5 (4.2, 10.8)	10.2 (6.2, 14.4)	2.7 (-2.1, 7.6)	36	0.3
nPCR, g/kg	0.97 (0.9, 1.06)	1.02 (0.94, 1.09)	+0.05 (-0.08, 0.18)	5.1	0.4

Footnote: *The data are shown as median (IQRs). Abbreviations: OH, overhydration; CRP, C-reactive protein; nPCR, normalized protein catabolic rate.
